# Effect of *L. reuteri* on bowel movements in children aged 6 months to 4 years: A double-blind randomized controlled trial

**DOI:** 10.3389/fped.2022.997104

**Published:** 2022-10-26

**Authors:** Camille Jung, Nicolas Kalach, Vanessa Degas, Yasmine Jeridi, Valérie Bertrand, Marc Bellaiche

**Affiliations:** ^1^ Clinical Research Center, Centre Hospitalier Intercommunal de Créteil, Créteil, France; ^2^ Department of Pediatrics, Groupement des Hôpitaux de L’Institut Catholique de Lille, Lille, France; ^3^ Department of Pediatrics, Centre Hospitalier Sud Francilien, Évry, France; ^4^ Department of Pediatrics, Service de Gastroenterologie et Hépatologie Pédiatriques- CHU de Toulouse, Toulouse, France; ^5^ Department of Pediatrics, Groupe Hospitalier du Havre, Le Havre, France; ^6^ Department of Pediatric Gastroenterology, Hôpital Robert Debré (AP-HP), Paris, France

**Keywords:** functional constipation, *Limosilactobacillus reuteri*, children, infant, randomized controlled trial

## Abstract

**Background:**

Chronic constipation is common in children and often requires prolonged laxative treatment. Preliminary studies suggest that the probiotic *Limosilactobacillus reuteri* (*L. reuteri*) may be useful in treating constipation in children, but these preliminary results need to be replicated. The objective of this study was to assess the efficacy of *L. reuteri* in infants and young children with chronic functional constipation.

**Methods:**

A prospective double-blind randomized placebo-controlled trial was conducted in 5 pediatric departments in France between June 2017 and June 2021. In all, 49 patients—ages 6 months to 4 years, and suffering from chronic constipation per Rome IV criteria—were randomly allocated to the test and control groups. For 4 weeks, all were orally administered 5 daily drops of the test (*L. reuteri* DSM 17938 at 10^8^ colony-forming units per day) or control (placebo) treatment, respectively. Participants were clinically assessed at 4 and 8 weeks. Parents were asked to daily record the number of spontaneous bowel movements (SBMs), stool consistency, and the use of any additional laxatives. Informed consent was obtained from parents of all recruited patients, and the study was approved by both an ethics committee and the French National Agency for Medicines and Health Products Safety (ANSM). The study is registered on ClinicalTrials.gov (NCT03030664).

**Results:**

The change in SBMs relative to baseline was greater in the control group at week 4 (control: 0.27 ± 0.5; test: 0.23 ± 0.5; *P* = 0.01) and in the test group at week 8 (control: 0.26 ± 0.4; test: 0.22 ± 0.5; *P* = 0.03). At week 4, the groups did not differ in number of responders (≥3 stools per week, with no non-retentive fecal incontinence), use of rescue medication, scoring of pain during defecation (Faces Pain Scale–Revised), or stool consistency (Bristol Stool Form Scale).

**Conclusion:**

This double-blind randomized controlled trial did not confirm the efficacy of *L. reuteri* for treatment of chronic functional constipation in young children.

## Introduction

Constipation, the infrequent, difficult, and painful passage of hard or sometimes very large stools, is one of the most common functional gastrointestinal (GI) disorders in the pediatric population. It is often associated with abdominal pain in children. Childhood constipation, which lowers patients’ quality of life, is a very frequent complaint in pediatric gastroenterology, accounting for a quarter of all appointments (1). In >90% of pediatric cases, constipation is functional (i.e., idiopathic), as defined by Rome IV criteria (2, 3). The prevalence of pediatric chronic functional constipation (CFC) is as high as 30% in Western nations (1).

GI motility is a complex mechanism affected by behavior (e.g., retentive postures and stool withholding in infants and children) and diet. Accordingly, recommended treatments combine lifestyle and dietary changes with use of oral laxatives. Macrogol, also known as polyethylene glycol (PEG), is the preferred laxative for pediatric CFC (1). Macrogol polymers bind water molecules within the colonic lumen. This increases the volume of water within the intestinal lumen, exerting a laxative effect. The efficacy of oral laxatives, and especially macrogol, has been established, but relapses after discontinuing their use are common (4).

The gut microbiota also plays a role in GI motility: some studies have emphasized that gut microbiota differed between patients with functional GI disorders and control subjects (5). *Lactobacilli* in particular may prevent or alleviate CFC by promoting intestinal peristalsis through the production of lactic acid and acetic acid, thereby increasing stool moisture (6). Jomehzadeh et al. recently showed that the gut flora of constipated children contained fewer lactobacilli (6).

Lactobacilli are gram-positive rod-shaped bacteria of the phylum *Firmicutes*. In 2020, the 261 known species of *Lactobacillus* were regrouped into 25 genera to account for their great diversity. *Lactobacillus reuteri* thus became *Limosilactobacillus reuteri* (*L. reuteri*). *L. reuteri* belongs to the obligately heterofermentative group of species previously in the genus *Lactobacillus*. Members of this group always ferment carbohydrates, generating lactic acid, ethanol, acetic acid, and carbon dioxide as by-products (7). Several comparative studies on the efficacy of *L. reuteri* for CFC in infants and young children have been conducted, yielding mixed results. In 2010, Coccorullo et al. published findings from a double-blind randomized controlled trial (RCT) that included 22 infants 5–10 months old treated with *L. reuteri* [10^8^ colony-forming units per day (CFU/day)] or placebo. The authors reported an increase in bowel movement frequency at weeks 4 and 8 for the *L. reuteri* group but no improvement in stool consistency or decrease in crying episodes (8). Similarly, Kubota et al. showed that bowel movement frequency had increased after 2 and 4 weeks of treatment with magnesium oxide (MgO), *L. reuteri*, or both (combination therapy); however, at week 4, Bristol Stool Form Scale scores had not improved in the probiotic group (9). In contrast, Wegner et al. detected no improvement of severe CFC in children aged 3–7 after administration of *L. reuteri* in combination with macrogol (10). Yet it is possible that their negative results reflect the interference by macrogol of *L. reuteri* interactions with the enterocyte membrane (11–13).

We sought to assess the efficacy of *L. reuteri* DSM 17938 as monotherapy for constipation in infants and young children.

## Materials and methods

### Design and participants

Our study was a prospective, multicenter, double-blind, placebo-controlled RCT with two-arm parallel assignment that ran from June 2017 to June 2021. The study was approved by an ethics committee (CPP IDF III; ref.: Am7650-2-3442); authorized by the French National Agency for Medicines and Health Products Safety (ANSM; no.: 2016071300014); and registered on the ClinicalTrials.gov website (NCT03030664). For recruitment, the study was presented during children's appointments with pediatric gastroenterologists at any of the 5 participating hospitals. The children were examined and their eligibility to participate was determined. This constituted the screening stage.

Children could be included if they suffered from CFC as defined by Rome IV (3), had a stable diet, and were available throughout the study period. They were excluded if they had a severe chronic disease; were suspected food intolerance; had undergone an operation for a GI problem during the year preceding inclusion; had taken *L. reuteri*, probiotics, or antibiotics during the 15 days preceding randomization; or had severe constipation unresponsive to 3 months of properly administered therapy.

After time for reflection, if the parents (or legal guardians) of a child agreed to the latter's participation, they provided their written informed consent. In that case, they were provided with diaries in which, on a daily basis throughout the duration of the study, parents were asked to report their child's bowel movements, record observations of stool consistency and pain symptoms, and note any other treatments administered. Parents were also asked to administer a laxative enema before randomized allocation, which was scheduled to take place after the run-in period (10–14 days) that followed the screening appointment. Randomization was performed at a ratio of 1:1 using an interactive online system for double-blind masking. The randomization code was not shared with subjects, study personnel, or the study sponsor. After randomization, parents were contacted by phone at week 2, to be informed of any side effects, verify treatment compliance, and ensure they were recording data in their diaries. Their children were called in for appointments again at weeks 4 and 8.

### Test treatment

For 4 weeks, test group subjects received 5 drops daily (10^8^ CFU/day) of an oil suspension containing *L. reuteri* DSM 17938 dispensed from a dropper bottle. The suspension was prepared by adding freeze-dried *L. reuteri* to a mixture of pharmaceutical-grade medium-chain triglycerides, sunflower oil, and pharmaceutical-grade silicon dioxide, to ensure adequate rheological properties (14). The placebo lacked only *L. reuteri*: its packaging and preparation was otherwise identical to the test treatment. After the 4-week treatment period, patients were followed up for another 4 weeks (i.e., until 8 weeks after the screening appointment).

### Additional treatments allowed during study

During the first week of the run-in period, no laxative treatment was administered. At the end of the run-in period, parents were asked to administer to their children an enema containing sodium citrate dihydrate and sodium lauryl sulfoacetate (Microlax), for children <18 months old, or sodium dihydrogen phosphate dihydrate and sodium hydrogen phosphate dodecahydrate (Normacol), for those ≥18 months old.

Lactulose (Duphalac) rescue medication was authorized during the study period, at a dosage of 5 ml/day for children <18 months old and 10 ml/day for those ≥18 months old, if ≥5 days had elapsed without a bowel movement.

### Outcome measures

The primary outcome measure was the difference in the number of spontaneous bowel movements (SBMs) per week, between week 4 (i.e., after randomization and treatment) and baseline (i.e., before randomization). The secondary outcome measures were the number of SBMs during week 8; the number of responders during week 4 (≥3 SBMs during week); stool consistency per the Bristol Stool Form Scale (15); level of pain during defecation, which was assessed by parents with the Faces Pain Scale–Revised (FPS-R), routinely used in pediatric settings (16, 17); how often children were administered conventional laxative treatment as rescue medication; and PedsQL scores, measuring the quality of life of children and their parents (https://www.pedsql.org/).

Stools were considered “hard,” indicative of constipation, if they were of Bristol Stool Form Scale Type 1 or 2; of “normal” consistency if of Type 3 or 4; and “liquid” if of Type 5–7. Pain ratings were “no pain” (FPS-R score: 0 or 2), “moderate” (FPS-R score: 4 or 6), and “severe” (FPS-R score: 8 or 10). For the PedsQL Family Impact Module, we applied the scoring procedure of the module's authors (https://www.pedsql.org/).

### Sample-size analysis

In light of a study by Ojetti et al. (18), we assumed that the difference in primary outcome measures (i.e., increase in number of SBMs per week) between the test and control groups would be 1.00. For a two-tailed alpha level of 0.05, a power of 90%, and a standard deviation of 1.07 for the primary outcome measure, 24 subjects per arm were needed. With a 20% of lost of follow-up or primary endpoint not available, it was planned to recruit 58 subjects.

### Statistical analyses

All data were saved in a database shared by all study centers and analyzed using version 9.4 of SAS statistical software (SAS Institute, Cary, NC, USA). For each treatment group, a descriptive analysis of initial patient characteristics was performed. Qualitative variables are described by numbers and percentages; quantitative variables, by means and standard deviations. All subjects with available bowel habit questionnaire were analyzed within their treatment groups. In case of missing data on the primary outcome, imputation on the mean have been applied. All *P* values under 0.05 were deemed significant. Depending on the distribution, either Student's *t*-test or the Mann-Whitney test were used to compare numbers of SBMs in the two groups. Proportions were compared using chi-square and Fisher's exact tests. Finally, adverse events were described in subjects who actually received the treatment.

## Results

Between June 2017 and June 2021, 49 children aged 6 months to 4 years and suffering from CFC by Rome IV criteria were included in our study. Inclusions were interrupted for 3 months between July 1 and October 1, 2019, due to a temporary treatment shortage. During the first COVID-19 lockdown in France, running from March to June 2020, follow-up continued for patients already included, but no new participants could be recruited as nonurgent medical appointments were not authorized.

**Figure 1:**
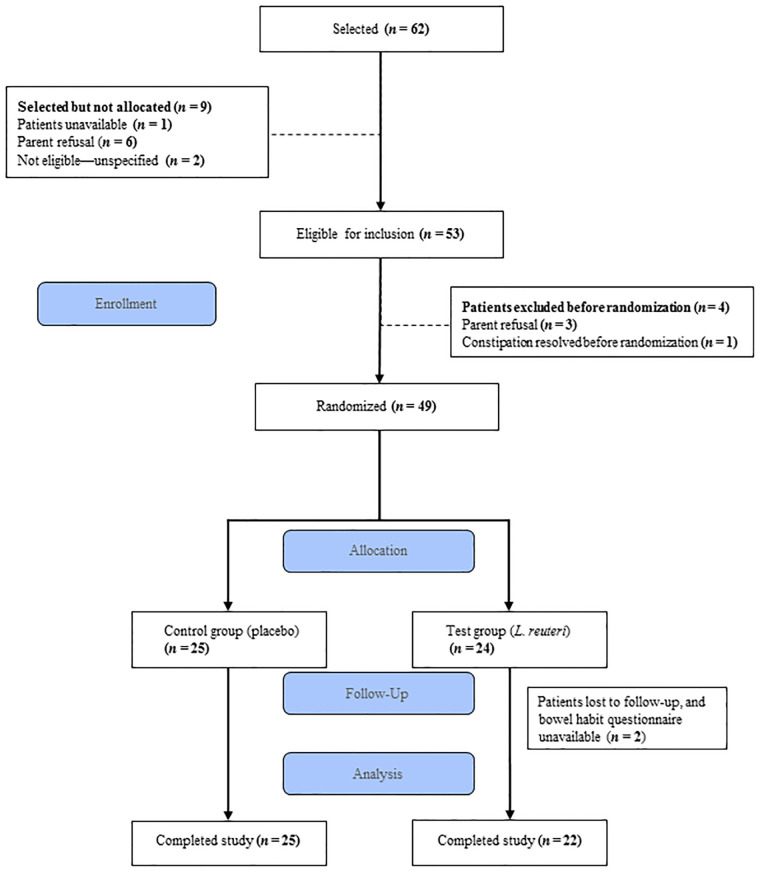
Study flow diagram.

In all, 62 patients were initially selected, 53 passed screening and had parents who consented to their participation, and 49 were randomized: 25 assigned to the control group (placebo) and 24 to the test group (*L. reuteri*). Two patients in the test group were lost to follow-up, leaving 22 who could be analyzed (Figure 1). Because patients were recruited during consultations for different reasons, the total number of patients between 6 months and 4 years of age with constipation seen by the investigators was not available. Both the test and control groups were similar in terms of age, sex, and baseline clinical characteristics such as height and weight (Table 1).

**Table 1:** Baseline demographics.

Characteristics	Total (*N* = 47)	*L. reuteri* (*n* = 22)	Placebo (*n* = 25)	P value
n (% of total)	47 (100.0)	22 (46.8)	25 (53.1)	
Female, n (%)	22 (46.8)	9 (40.9)	13 (52.0)	0.44^a^
Male, n (%)	25 (53.1)	13 (59.0)	12 (48.0)	
Age in months, mean (±SD)	28.1 (10.8)	28.7 ± 9.8	27.7 ± 11.7	0.75^b^
Weight in kg, mean (±SD)	13.2 (2.9)	13.1 ± 2.8	13.3 ± 3.1	0.80^b^
Height in cm, mean (±SD)	90.1 (10.3)	88.6 ± 9.3	91.4 ± 11.2	0.36^b^

SD, standard deviation.

^a^ chi-square test.

^b^ Student's *t*-test.

**Figure 2:**
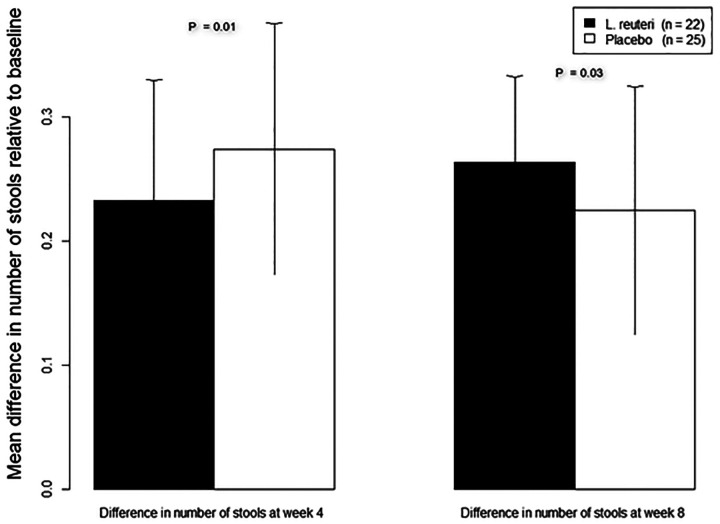
Change in number of stools at weeks 4 and 8 relative to baseline.

**Figure 3:**
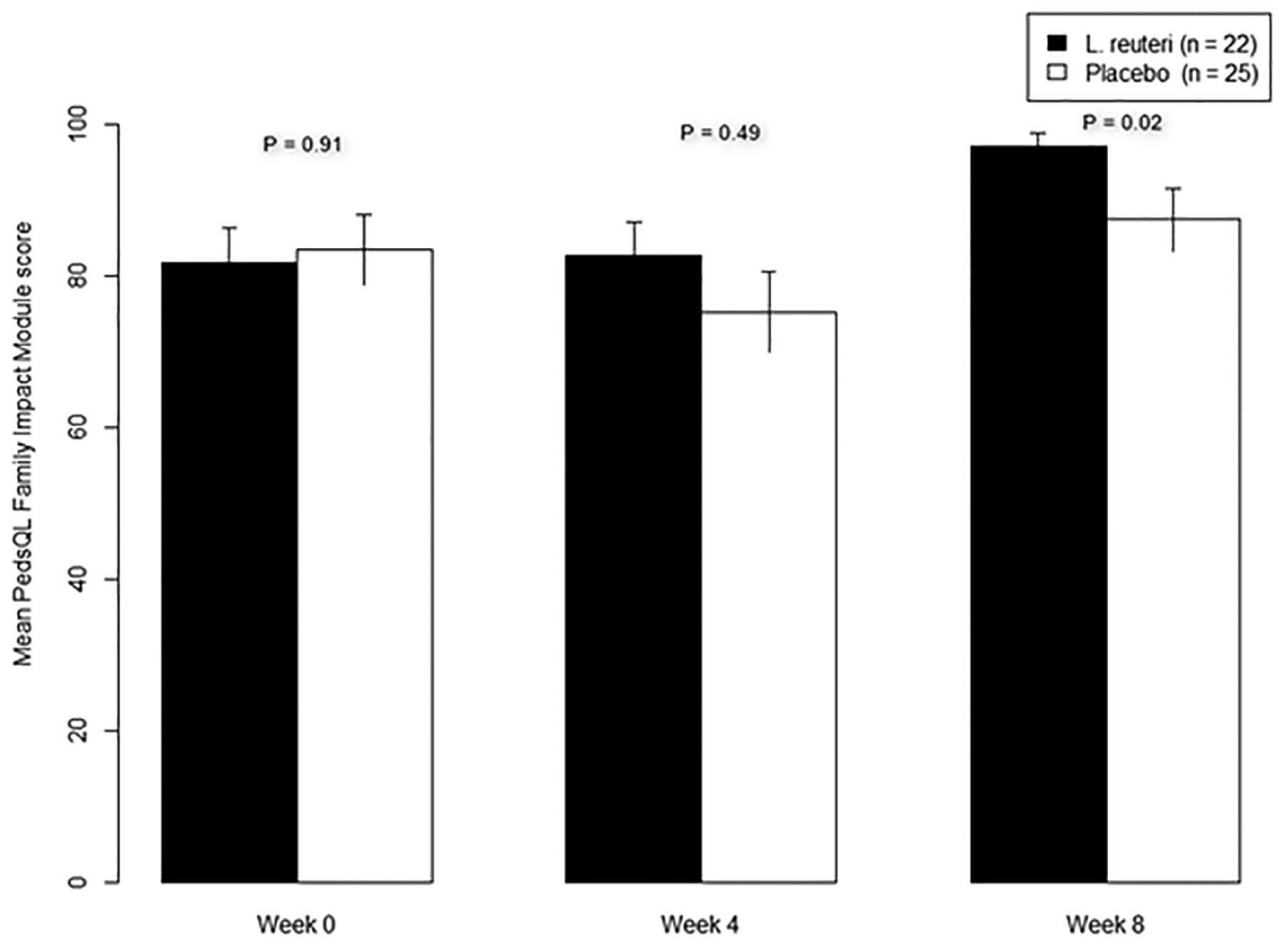
Change in PedsQL family impact module scores for test and control groups.

The number of SBMs per week increased for both groups at weeks 4 and 8. The increase in number of SBMs per week with respect to baseline was significantly higher for the control group at week 4 (0.27 ± 0.5, *P* = 0.01) and for the test group at week 8 (0.26 ± 0.4, *P* = 0.03) (Figure 2). The groups did not differ in numbers of responders (i.e., ≥3 stools per week, without fecal incontinence) at week 4 (test: 18/22, 82%; control: 22/25, 88%; *P* = 0.55). In both groups, rescue medication was used at similarly high frequencies (test: 16/22, 73%; control: 15/25, 60%; *P* = 0.35). Both groups exhibited similar Bristol stool consistencies and pain levels (rated by parents using FPS-R) at baseline and week 4 (Table 2). PedsQL Family Impact Module scores were identical in both study arms at baseline and week 4, while the test group scored higher than the control group at week 8 (97.4 ± 9 in the test group vs. 87.4 ± 20.9 in the control group, *P* = 0.028, Figure 3).

**Table 2:** Stool consistency and pain assessment at baseline and week 4.

Group	Weeks	Total (*N* = 47)	*L. reuteri* (*n* = 22)	Placebo (*n* = 25)	P value
Stool consistency					
Hard, n (%)	0	32 (68.08)	13 (59.0)	19 (53.1)	0.22^a^
Normal, n (%)		2 (4.25)	2 (9.0)	0 (0.0)	
Liquid, n (%)		13 (27.65)	7 (31.8)	6 (24.0)	
Hard, n (%)	4	15 (31.91)	9 (40.9)	6 (24.0)	0.33^a^
Normal, n (%)		10 (21.27)	3 (13.6)	7 (28.0)	
Liquid, n (%)		22 (46.80)	10 (45.4)	12 (48.0)	
Pain assessment					
No pain, n (%)	0	0 (0.0)	0 (0.0)	0 (0.0)	0.06^a^
Moderate pain, n (%)		19 (40.4)	12 (54.5)	7 (28.0)	
Severe pain, n (%)		28 (59.5)	10 (45.5)	18 (72.0)	
No pain, n (%)	4	13 (31.7)	4 (22.2)	9 (39.1)	0.36^a^
Moderate pain, n (%)		20 (48.8)	9 (50.0)	11 (47.8)	
Severe pain, n (%)		8 (19.5)	5 (27.7)	3 (13.0)	

^a^ Fisher's exact test.

No serious adverse event was reported. In the test group, 4 nonserious adverse events were reported: 1 episode of intense abdominal pain during defecation, 1 episode of anal bleeding during defecation, 1 episode of diarrhea, and 1 nasopharyngitis (Supplementary Table S1).

## Discussion

In this placebo-controlled RCT, we assessed the efficacy of *L. reuteri* DSM 17938 as a monotherapy for CFC in infants and young children. SBM frequency and stool consistency was no greater in the test group than among controls after 4 weeks of treatment, but SBM frequency and quality of life were higher in the test group than among controls after 8 weeks.

Our study was similar in design to the study published in 2014 by Ojetti et al., who considered 40 adults with CFC per Rome III criteria. However, they reported greater improvements with *L. reuteri* treatment: after 4 weeks of treatment, the mean number of SBMs per week was higher among test subjects (5.3) than controls (3.9), though stool consistencies did not differ between groups (18). These findings are comparable to those published by Coccorullo et al. (8) for CFC in infants. Their double-blind placebo-controlled RCT tested *L. reuteri* at the same dosage we used (10^8 ^CFU/day) but over a longer treatment period: 8 weeks, rather than our 4 weeks. Coccorullo et al. reported significant improvements in the number of weekly SBMs within the test group, from week 2 through week 8, but none in stool consistency or crying during bowel movements. García Contreras and colleagues (19) recently reported on their analysis of 30 infants with cerebral palsy randomly allocated to one of three groups, for probiotic (*L. reuteri*), prebiotic, or combination treatment. In their study, frequency of normal stools did not improve in the probiotic group, despite decreased stool pH levels and increased stool *L. reuteri* concentrations. These data, like those published earlier (8, 18), corroborate our own, which show no improvement in stool consistency with *L. reuteri* treatment.

The findings of Kubota et al. (9) published in 2020 are, however, less clear-cut. They considered 3 experimental groups, respectively treated with MgO and placebo; MgO and *L. reuteri*; and *L. reuteri* and placebo. SBM frequency increased between weeks 0 and 4 for all groups, but MgO was most effective for softening stools. Combination therapy exhibited no synergistic effect.

Other authors have considered the value of using probiotics as an adjuvant to standard laxative treatment. Wegner et al. (10) conducted a multicenter, double-blind RCT assessing the efficacy of *L. reuteri* as an adjuvant to macrogol in 121 children ages 3–7 with severe functional constipation refractory to conventional treatment (macrogol, lactulose, MgO, mineral oil, or enemas) administered for ≥2 months before inclusion. They observed an increase in the number of SBMs in both the test and control groups, though there was no statistical difference between groups. There was also no significant difference in numbers of responders (i.e., ≥3 SBMs a week).

We chose not to combine *L. reuteri* with macrogol (i.e., PEG) in our study because it has been shown that the latter can alter the action of *L. reuteri* at the enterocyte membrane. Lactulose significantly changes the composition of fecal flora; PEG, on the other hand, inhibits most metabolic activities of that flora, reducing the amount of short-chain fatty acids, acetate, and butyrate, and decreasing fecal bacterial mass (11). Still, whether because they had not defecated for several days or because their parents believed their condition to be too painful, >60% of the subjects in our study were administered a laxative rescue medication. This rescue medication was the same for both arms, which suggests the absence of a synergistic effect when *L. reuteri* is combined with a laxative. It is also possible that the effects of concomitant laxatives could impact the activity of the probiotic. It should be recalled that the study by Wegner et al. included patients with severe constipation. In contrast, we sought to exclude patients with intractable constipation, i.e., unresponsive after ≥3 months of a properly administered laxative treatment. Yet our patients were recruited in hospitals, not primary care physicians’ offices, and most of those included had already been receiving follow-up care for constipation before their inclusion.

Another strategy is to prevent these GI disorders. Thus, in an RCT whose findings were published in 2014, Indrio et al. (20) observed that stool frequency at 1 and 3 months was higher in infants preventively administered *L. reuteri* DSM 17938 than in subjects given the placebo treatment. Thus, early intervention to promote gut colonization by lactobacilli during the first weeks of life could lower the incidence of functional constipation in young children, and it may be more effective to adopt a strategy of rapid treatment before weeks of constipation and stool-withholding behavior elapse.

It is difficult to conduct RCTs that compare placebos to products already available over the counter. Nevertheless, there is a demand among parents of children with CFC for alternatives to conventional laxative treatments, administered over shorter periods than the latter. Our assessment of the efficacy of 4-week *L. reuteri* monotherapy was a methodological strength of this study. The initial target was 48 patients with questionnaire available. A total of 47 patients could be analyzed, a number that allowed us to detect a difference of 1 stool/week between the 2 groups with a power of 87%. However, a weak point of this study was parents’ frequent recourse to laxative rescue medication for their children.

In conclusion, this RCT did not clearly demonstrate the efficacy of *L. reuteri* for CFC in infants and young children.

## Data Availability

The raw data supporting the conclusions of this article will be made available by the authors, without undue reservation.
