# Supplementary material for: Effect of L. reuteri on bowel movements in children aged 6 months to 4 years: A double-blind randomized controlled trial
Source: Front Pediatr. 2022 Oct 26;10:997104. doi: 10.3389/fped.2022.997104 (PMC9643683; doi:10.3389/fped.2022.997104)
Supplement: Supplementary file 1 [file Table1.docx]

Supplementary Material

# Supplementary Table

***Supplementary Table 1. Adverse events.***

| Characteristics | Total (*N* = 47) | *L. reuteri* (*n* = 22) | Placebo  (n=25) | P-value* |
| --- | --- | --- | --- | --- |
| n (%) | 47 (100.0) | 22 (46.8) | 25 (53.1) |  |
| Number of children with AEs | 11 | 3 (27.2) | 8 (72.3) | 0.18 |
| Number of AEs | 13 | **4**/13 | **9**/13 |  |
| Miscellaneous AEs |  |  | **1** |  |
| Finger injury |  |  | 1 |  |
| GI AEs |  | **3** | **3** |  |
| Pain during emission |  | 1 |  |  |
| Fecal impaction |  |  | 1 |  |
| Anal bleeding |  | 1 | 1 |  |
| Diarrhea |  | 1 |  |  |
| Abdominal pain and uncontrollable bowel movements |  |  | 1 |  |
| Infectious AEs |  | **1** | **5** |  |
| Cough |  |  | 1 |  |
| Fever |  |  | 1 |  |
| Nasopharyngitidis |  | 1 |  |  |
| Tracheitis suggestive of viral etiology |  |  | 1 |  |
| Tonsillitis |  |  | 1 |  |
| Gastroenteritis |  |  | 1 |  |

*, Fisher’s exact test

AE, adverse event; GI, gastrointestinal.
