# Supplementary material for: Effect of L. reuteri on bowel movements in children aged 6 months to 4 years: A double-blind randomized controlled trial
Source: Front Pediatr. 2022 Oct 26;10:997104. doi: 10.3389/fped.2022.997104 (PMC9643683; doi:10.3389/fped.2022.997104)
Supplement: Supplementary file 2 [file Table2.docx]

Supplementary Material

***Supplementary Table 2. Rescue medications.***

|  |  | *L. reuteri* (*n* = 22) | Placebo  (n=25) | P-value* |
| --- | --- | --- | --- | --- |
| n (%) |  | 22 (46.8) | 25 (53.1) |  |
| Number of children with additional laxative treatment | | 16 (72.7) | 15 (60.0) | 0.35 |
| Method of administation | **Types of laxatives** |  |  |  |
| Oral | Lactulose | 7 (31.8) | 6 (24.0) | 0.80 |
|  | Macrogol | 1 (4.5) | 0 (0) |  |
|  | Paraffin oil | 1 (4.5) | 0 (0) |  |
| Enema | Microlax® (sodium lauryl sulfoacetate) | 4 (18.2) | 4 (16.0) |  |
|  | Normacol® (phosphate monosodique dihydrate/phosphate disodique dodécahydrate) | 1 (4.5) | 4 (16.0) |  |
| Suppository | Glycerin suppository | 0 (0) | 1 (4.0) |  |
| Enema and oral medication | Glycerin suppository and Microlax®  Normacol® and Lactulose | 1 (4.5)  1 (4.5) | 0 (0)  0 (0) |  |

*, Fisher’s exact test
