# Supplementary material for: Effect of L. reuteri on bowel movements in children aged 6 months to 4 years: A double-blind randomized controlled trial
Source: Front Pediatr. 2022 Oct 26;10:997104. doi: 10.3389/fped.2022.997104 (PMC9643683; doi:10.3389/fped.2022.997104)
Supplement: Supplementary file 3 [file Table3.docx]

Supplementary Material

***Supplementary Table 3. Mean number of medication doses for each laxative.***

|  | Mean of doses per medication , Mean ±sd | | P-value |
| --- | --- | --- | --- |
| Type of laxative | **Test treatment**  **(N=22)** | **Placebo**  **(N=25)** |  |
| Lactulose | 7.6±8.5 | 17.1±11.6 | 0.13 |
| Macrogol | 16 |  |  |
| Paraffin oil | 4 |  |  |
| Normacol® (phosphate monosodique dihydrate/phosphate disodique dodécahydrate) | 5±2.8 | 3±3.4 | 0.34 |
| Microlax® (sodium lauryl sulfoacetate) | 5±2.3 | 4.5±2.0 | 0.88 |
| Glycerin suppository |  | 12 |  |

^: WUM test
